# Comparison of dual-bolus versus dual-sequence techniques for determining myocardial blood flow and myocardial perfusion reserve by cardiac magnetic resonance stress perfusion: From the Automated Quantitative analysis of myocardial perfusion cardiac Magnetic Resonance Consortium

**DOI:** 10.1016/j.jocmr.2024.101085

**Published:** 2024-08-16

**Authors:** Emily Yin Sing Chong, Haonan Wang, Kwan Ho Gordon Leung, Paul Kim, Yuko Tada, Tsun Hei Sin, Chun Ka Wong, Kwong Yue Eric Chan, Chor Cheung Frankie Tam, Mitchel Benovoy, Andrew E. Arai, Victor Goh, Martin A. Janich, Amit R. Patel, Ming-Yen Ng

**Affiliations:** a Department of Diagnostic Radiology, School of Clinical Medicine, Li Ka Shing Faculty of Medicine, The University of Hong Kong, Hong Kong; b GE HealthCare, Waukesha, Wisconsin, USA; c Division of Cardiovascular Medicine, Department of Medicine, University of California San Diego, La Jolla, California, USA; d Division of Cardiology, Department of Medicine, Queen Mary Hospital, Hong Kong; e Cardiology Division, Department of Medicine, School of Clinical Medicine, Li Ka Shing Faculty of Medicine, the University of Hong Kong; f McGill University, Montreal, Quebec, Canada; g Division of Cardiovascular Medicine, Department of Medicine, University of Utah School of Medicine, Salt Lake City, Utah, USA; h Hong Kong Sanatorium & Hospital, Hong Kong; i GE HealthCare, Munich, Germany; j Division of Cardiovascular Medicine, The University of Virginia Health System, Charlottesville, Virginia, USA

**Keywords:** Quantitative stress perfusion, Dual bolus, Dual sequence, Myocardial blood flow, Myocardial perfusion reserve, Cardiac magnetic resonance

## Abstract

**Background:**

Quantitative stress cardiac magnetic resonance (CMR) can be performed using the dual-sequence (DS) technique or dual-bolus (DB) method. It is unknown if DS and DB produce similar results for myocardial blood flow (MBF) and myocardial perfusion reserve (MPR). The study objective is to investigate if there are any differences between DB- and DS-derived MBF and MPR.

**Methods:**

Retrospective observational study with 168 patients who underwent stress CMR. DB and DS methods were simultaneously performed on each patient on the same day. Global and segmental stress MBF and rest MBF values were collected.

**Results:**

Using Bland-Altman analysis, segmental and global stress MBF values were higher in DB than DS (0.22 ± 0.60 mL/g/min, p < 0.001 and 0.20 ± 0.48 mL/g/min, p = 0.005, respectively) with strong correlation (r = 0.81, p < 0.001 for segmental and r = 0.82, p < 0.001 for global). In rest MBF, segmental and global DB values were higher than by DS (0.15 ± 0.51 mL/g/min, p < 0.001 and 0.14 ± 0.36 mL/g/min, p = 0.011, respectively) with strong correlation (r = 0.81, p < 0.001 and r = 0.77, p < 0.001). Mean difference between MPR by DB and DS was −0.02 ± 0.68 mL/g/min (p = 0.758) for segmental values and −0.01 ± 0.49 mL/g/min (p = 0.773) for global values. MPR values correlated strongly as well in both segmental and global, both (r = 0.74, p < 0.001) and (r = 0.75, p < 0.001), respectively.

**Conclusion:**

There is a very good correlation between DB- and DS-derived MBF and MPR values. However, there are significant differences between DB- and DS-derived global stress and rest MBF. While MPR values did not show statistically significant differences between DB and DS methods.

## Graphical abstract

**Figure:**
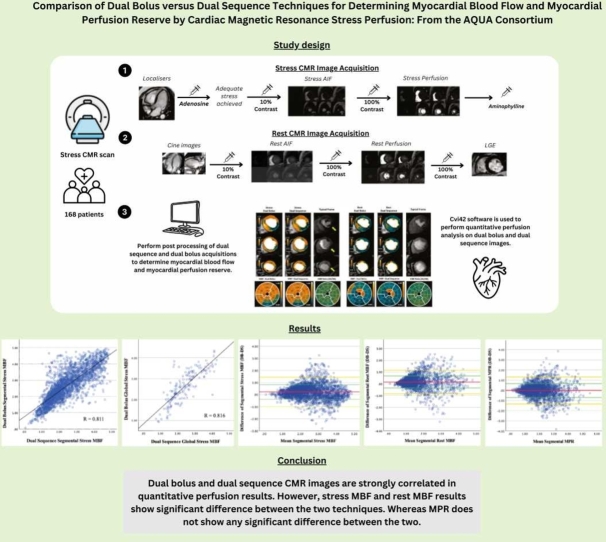

## 1 Introduction

Quantitative stress cardiovascular magnetic resonance (CMR) has been utilized to diagnose coronary artery disease and microvascular dysfunction [1], [2], as well as predict major adverse cardiovascular events [3].

Two methods have been utilized to perform quantitative stress CMR: dual sequence (DS) and dual bolus (DB). DS utilizes a magnetic resonance imaging (MRI) pulse sequence with a low spatial resolution spoiled gradient echo with a short-saturation preparation time in the basal region followed by three standard high-resolution perfusion images acquired in the short-axis oblique position of the left ventricular (LV) base, mid-ventricle, and apex. Advantages of this method include easy deployment for use without any significant changes in examination workflow. However, the DS is only available as “works in progress” and not all sites have access to this sequence.

DB requires the injection of a 10% contrast dilution bolus before the injection of the 100% contrast bolus. The perfusion sequence is then acquired continuously during the 10% contrast dilution injection followed by the 100% contrast injection. A software package to analyze the perfusion sequences and to determine the myocardial blood flow (MBF) and myocardial perfusion reserve (MPR) values is then required. These software packages are sold by various third-party vendors. DB is therefore available to any unit with an MRI scanner but it requires suboptimal workflow.

With quantitative stress CMR being increasingly utilized, it is unknown if DS and DB methods produce similar results for MBF and MPR. This could create discrepancies in our interpretation of these results. Hence, the objective of this study is to investigate if there are any differences between DB and DS methods in analyzing quantitative perfusion (QP) results.

## 2 Methods

This retrospective study received approval from Hong Kong West Cluster Institutional Review Board. Ethics approval number: UW24-197.

### 2.1 Study population

Conducted at the University of Hong Kong (HKU) MRI unit, this was a retrospective observational study with 204 patients who had their CMR scans performed between January 2022 and December 2022. Both DB and DS techniques were conducted on the same patient and on the same day of their CMR scan.

**Fig. 1:**
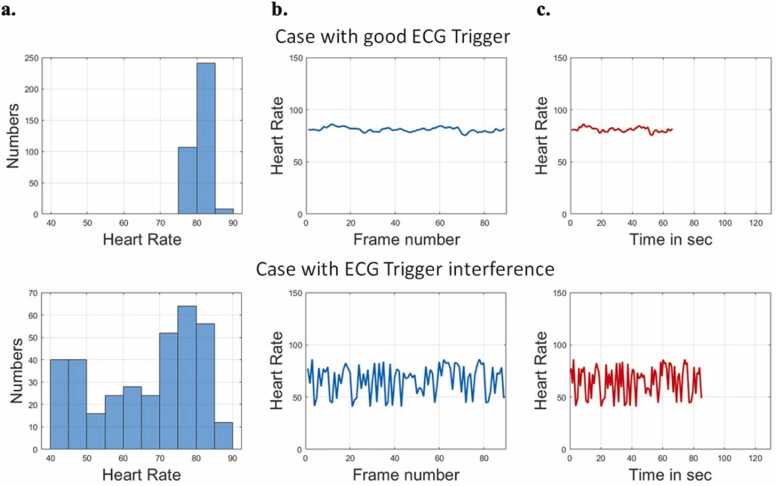
Cases with good ECG trigger and ECG trigger interference. Upper row is the quality check result of a good acquisition. Calculated heart rate is relatively stable across the whole acquisition. The diagrams from left to right are (a) histogram of heart rate during the acquisition, (b) heart rate vs frame number, and (c) heart rate vs time. Bottom row is an example of a case that is excluded from the analysis. The heart rate varies drastically across the acquisition, which indicates that this is a non-ideal ECG trigger. *ECG* electrocardiogram

Medical information and drug history were reviewed using the Electronic Patient Record. Patients 18 years old and above who underwent quantitative stress CMR scans using adenosine from the HKU MRI unit between January 1, 2022 and December 31, 2022 were included in the study. We excluded patients with (i) inadequate stress response during CMR scans, (ii) poor quality CMR images due to motion or strong electrocardiogram (ECG) interference that led to inconsistent cardiac phases (Fig. 1), (iii) incorrect DB protocol, (iv) congenital heart disease, (v) incomplete patient records or CMR scan, (vi) technical errors in QP of their CMR scans. Inadequate stress was defined as patients with heart rate rise ≤10 bpm during adenosine infusion, systolic blood pressure drop ≤10 mmHg, absence of splenic switch-off sign, and absence of symptoms (e.g., chest discomfort, shortness of breath). The final number of patients in this study was 168. Two patients with inadequate stress response during CMR scan were excluded. Twenty-one patients were excluded due to strong ECG interference that compromised QP quality (Fig. 1), four patients were excluded due to incorrect DB protocol (i.e., 0.05 mmol/kg [100%] gadoterate meglumine contrast was given before the 0.005 mmol/kg (10%) gadoterate meglumine contrast was injected), four patients were excluded due to incomplete CMR imaging. One patient was excluded due to incomplete patient records, two were excluded due to strong parallel image artifacts, one was excluded due to issues with measuring blood pool in a patient with hypertrophic cardiomyopathy, and one patient was excluded due to congenital heart disease diagnosis.

ECG interference was subject-dependent, often caused by factors such as breathing motion. An acquisition with ECG issues resulted in inconsistent cardiac phases, leading to motion correction failure, strong partial volume effects, and signal variation, which significantly impacted the MBF calculation. ECG waveform was checked based on the heart rate calculation in between the actual time point of adjacent frames.

### 2.2 CMR image acquisition

**Fig. 2:**
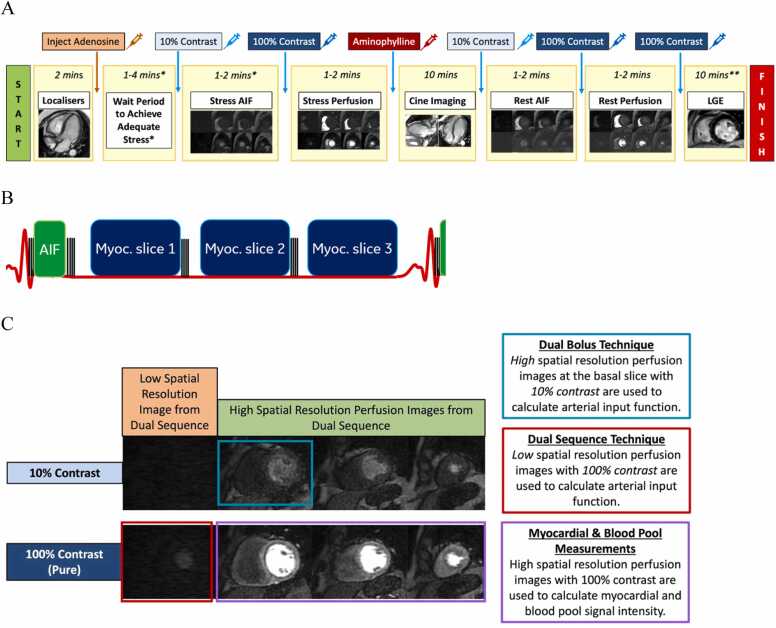
(a) Stress CMR imaging protocol which incorporates dual-bolus (DB) and dual-sequence (DS) techniques. (b) The DS CMR pulse sequence consists of low-resolution spoiled gradient echo with short-saturation preparation time in one basal short-axis slice for estimation of arterial input function (green box), B_1_-B_0_-robust saturation pulses (black box), and high-resolution and saturation-prepared myocardial imaging in 2–3 short-axis slices (blue box). (c) Both 0.005 mmol/kg (10%) gadoterate meglumine contrast and 0.05 mmol/kg (100%) gadoterate meglumine contrast acquisitions will have one low spatial resolution perfusion image produced at the basal slice and three high-resolution perfusion images at the basal, mid-ventricular, and apical slices. DB-derived stress/rest myocardial blood flow (MBF) and myocardial perfusion reserve (MPR) are determined by using the high-resolution perfusion images of the basal slice with 0.005 mmol/kg (10%) gadoterate meglumine contrast bolus. DS-derived stress/rest MBF and MPR are determined by using the low-resolution perfusion images of the basal slice with 0.05 mmol/kg (100%) gadoterate meglumine contrast bolus. Both DB and DS use the high-resolution perfusion images of the basal, mid-ventricular, and apical slices to calculate the myocardial and blood pool intensity to subsequently determine the stress/rest MBF and MPR. *CMR* cardiovascular magnetic resonance, *AIF* arterial input function, *LGE* late gadolinium enhancement

Patients were scanned using a SIGNA Premier 3.0T MRI scanner (GE Healthcare, Waukesha, Wisconsin) at the HKU MRI unit with a 32-element phased-array coil. A 140 mcg/kg/min of adenosine was infused in each patient for at least 3 min. Adenosine infusion concentration was increased to 210 mcg/kg/min and further infused for an extra 2 min if patient did not achieve a sufficient stress response. DB first-pass perfusion was then performed using multi-slice two-dimensional saturation recovery dual imaging sequence. The DS includes one low spatial resolution but high temporal resolution image from the basal short-axis slice for estimating the arterial input function (AIF) immediately following the R-wave trigger followed by three higher resolution short-axis images. To cover enough contrast wash-in and wash-out stages, 70 to 90 time frames were acquired. The proton density-weighted images were acquired as the first two frames of each slice for surface coil intensity correction. Other image parameters include field-of-view (FOV) = 32–38 × 24–28 cm, matrix size = 192 × 148, number of excitations (NEX) = 0.75, flip angle = 15, repetition time (TR)/echo time (TE) = 2.6–3.1/1.2–1.5 ms, and parallel imaging factor = 2. The CMR imaging acquisition protocol is shown below in Fig. 2 and Table 1.

**Table 1:** Stress perfusion protocol.

Scanner	3.0T SIGNA Premier
Contrast agent	Gadoterate meglumine: 0.05 mmol/kg
Flush	30 mL saline: 3 mL/s
Stress agent	Adenosine140 μg/kg/min over 3–4 min
Reversal agent	Aminophylline 75 mg
FOV	36–40 cm
Phase FOV	0.75–0.80
Matrix	192 × 148
Flip angle	20°
TR	3.4 ms
TE	1.1–1.5 ms
NEX	0.75
Slice thickness	8 mm

*FOV* field-of-view*, TR* repetition time*, TE* echo time*, NEX* number of excitations

### 2.3 Cardiac MRI image analysis

#### 2.3.1 Quantitative perfusion analysis

Images were analyzed at the HKU MRI unit. Prototype version of Circle Cardiovascular Imaging (cvi42 version 5.13.8, Circle Cardiovascular Imaging, Calgary, Alberta, Canada) was used.

**Fig. 3:**
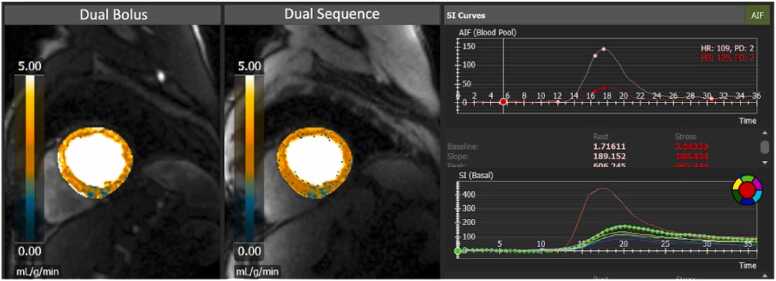
AIF curves and MBF curves for dual bolus and dual sequence. *AIF* arterial input function, *MBF* myocardial blood flow, *HR* heart rate, *SI* signal intensity, *PD* proton density.

**Fig. 4:**
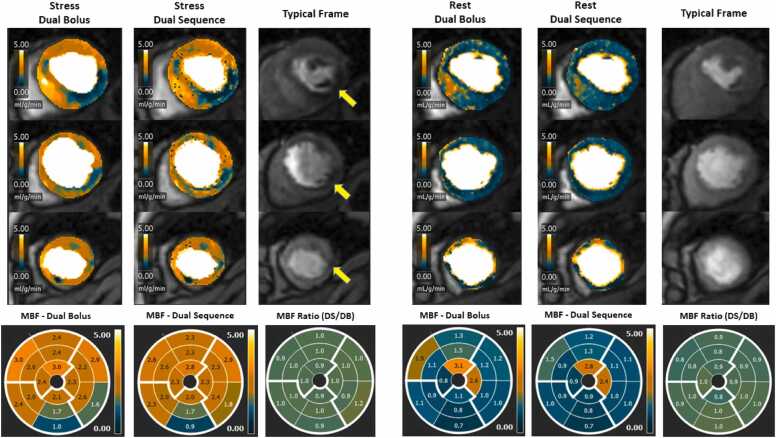
Dual-bolus (DB) dual-sequence (DS) case example. This is a case of a patient with obstructive coronary artery disease in the right coronary artery territory. First and second columns compare stress myocardial blood flow (MBF) between DB and DS. Fourth and fifth columns compare rest MBF between DB and DS. There is good agreement between different American Heart Association segments with minor differences. DB MBF seems overall slightly higher than DS. The MBF ratio plots are created by loading the stress DB and DS images into the module simultaneously allowing the software to generate a ratio

For QP analysis, contours of LV epicardium, LV endocardium, right ventricular (RV) endocardium, and insertion points were generated using cvi42 software. The operator (H.W.) is experienced in cvi42 software and was blinded to the patient data. Manual contour adjustments were made to increase the accuracy. The same contours were used between the DB and DS analysis to minimize potential bias (Fig. 3). Median stress MBF and rest MBF values for all 16 American Heart Association (AHA) segments were acquired from the system. Global stress MBF and global rest MBF were calculated using the average of the 16 AHA segmental values for the two respective parameters. Global MPR value for each patient was obtained based on the ratio of global stress MBF to global rest MBF (Fig. 4).

The method to extract AIF information with different scaling factors was based on a previously published method by Hsu et al.[10]. First, the impulse response function is normalized and transformed into a probability density function that describes the contrast transit times through the myocardial tissue. So, if the AIF and the myocardial perfusion images are on different but linear scales, converting to a probability density function adjusts for the differing scaling factors. The initial amplitude of this probability density function represents the blood flow through the region in response to the instantaneous arrival of the contrast agent.

#### 2.3.2 Wall thickness analysis

Wall thickness was suspected to be a potential factor in creating variances between DB and DS QP results. During QP image processing, it has been observed that myocardial wall thickness that is too thin could lead to anomalous values due to the effect of partial voluming. To determine if wall thickness had an impact on differences in DB and DS techniques, the wall thickness for each segment was measured. Images were analyzed at the HKU MRI unit with cvi42.

Stress images with 0.05 mmol/kg (100%) gadoterate meglumine contrast were used. Specific slice to measure wall thickness was chosen during the first pass, where RV and LV regions are well-opacified. Basal, mid-ventricular, and apical slices are identical to the slices in the QP analysis for each case. The epicardial and endocardial surfaces of basal, mid-ventricular, and apical slices were contoured using Function SAX. Wall thickness values were categorized into AHA segments. The average value of the wall thickness for each slice was calculated automatically and assigned to the corresponding AHA segments. The mean wall thickness value for AHA segments 1 to 6 represented the mean basal wall thickness. The mean wall thickness value for AHA segments 7–12 represented the mean mid-ventricular wall thickness. Last, AHA segments 13–16 wall thickness values were averaged to find the mean apical wall thickness.

### 2.4 Statistical analysis

IBM SPSS Statistics software (IBM Corporation, Armonk, New York) was utilized. Dual-bolus and dual-sequence techniques were compared by using the global stress MBF, global rest MBF, and global MPR values of each case. Bland-Altman plots for all three parameters were constructed, with the difference between DB and DS MBF or MPR values on the Y-axis, and the mean MBF or mean MPR values on the X-axis. Linear regression analysis was performed with DB parameters as the dependent variable and DS parameter as the independent variable. Beta coefficients for global stress MBF, global rest MBF, and global MPR were obtained. Pearson’s correlation was performed to analyze the strength of the correlations between DB and DS values of each parameter, and p-values were recorded.

Shapiro-Wilk’s test was performed for all three QP parameters at both global and segmental levels to determine whether or not data were normally distributed. As these values were not normally distributed, Mann-Whitney U test was performed to assess for significant differences between the QP parameters at both global and segmental levels.

A sub-analysis between wall thickness and differences between QP values (DB-DS) was performed to investigate if wall thickness (basal, mid-ventricular, and apical) could potentially create discrepancies in QP analysis or have different effects on DB and DS QP results. Pearson’s correlation was performed between wall thickness by slices and differences between DB and DS QP values for stress MBF, rest MBF, and MPR.

## 3 Results

### 3.1 Baseline patient demographics

Patient demographics, diagnosis, risk factors, CMR parameters and drugs are listed in Table 2. Our cohort had a mean age of 62.4 ± 12.6 years old, 96 patients (57.1%) were male and 72 patients (42.9%) were female. Sixty-four (38.1%) patients had known coronary artery disease (CAD) before their CMR scan. Of these 64 patients, 51 patients (30.4%) had obstructive CAD or ischemia on CMR and 117 (69.9%) had non-obstructive CAD or no ischemia on CMR. Three (1.8%) patients had hypertrophic cardiomyopathy and 12 (7.1%) patients had dilated cardiomyopathy. A small proportion of patients had arrhythmia. Four (2.4%) patients with atrial fibrillation and four (2.4%) with premature ventricular complexes (i.e., ectopic heartbeats).

**Table 2:** Demographics, clinical, and drug history of study population.

	N = 168
*Demographics*	
Male	96 (57.1%)
Female	72 (42.9%)
Age (years)	62.4 ± 12.6
Body mass index (kg/m^2^)	24.3 ± 4.2
*Diagnosis*	
Obstructive CAD/ischemia on CMR	51 (30.4%)
Non-obstructive CAD/no ischemia on CMR	117 (69.6%)
Hypertrophic cardiomyopathy	3 (1.8%)
Dilated cardiomyopathy	12 (7.1%)
*Risk factors*	
Atrial fibrillation	4 (2.4%)
PVC (ectopic)	4 (2.4%)
Perfusion defect	39 (23.2%)
Type 2 diabetes mellitus (DM2)	79 (47.0%)
Hypertension	92 (54.8%)
Dyslipidemia	100 (59.5%)
Smoking	38 (22.6%)
Obesity	40 (23.8%)
PCI before CMR scan	19 (11.3%)
CABG before CMR scan	2 (1.2%)
Family history of premature coronary artery disease (%)	15 (8.9%)
*Drugs*	
ACEI/ARB	89 (53.0%)
Beta blockers	56 (33.3%)
Calcium channel blockers	53 (31.5%)
Diuretics	24 (14.3%)
Clopidogrel	17 (10.1%)
Aspirin	57 (33.9%)
Statins	126 (75%)
Anti-diabetes	74 (44.0%)
Anti-coagulants	5 (3.0%)
Nitrates	35 (20.8%)
CMR	
Stress heart rate (bpm)	84.3 ± 14.3
Rest heart rate (bpm)	68.4 ± 12.3
LVEDVI (mL/m^2^)	81.2 ± 21.9
LVESVI (mL/m^2^)	35.5 ± 20.3
LVSVI (mL/m^2^)	45.7 ± 8.5
LVEF (%)	58.1 ± 10.5
LVMI (g/m^2^)	54.9 ± 14.1
RVEDVI (mL/m^2^)	78.6 ± 15.2
RVESVI (mL/m^2^)	33.1 ± 9.9
RVEF (%)	58.5 ± 7.1
LGE (positive)	42 (25%)
Ischemic LGE	21 (12.5%)
Non-ischemic LGE	24 (14.3%)

*CAD* coronary artery disease, *PVC* premature ventricular complex, *PCI* percutaneous coronary intervention, *CABG* coronary artery bypass graft, *CMR* cardiac magnetic resonance, *LVEDVI* left ventricular end-diastolic volume index, *LVESVI* left ventricular end-systolic volume index, *LVSVI* left ventricular stroke volume index, *LVEF* left ventricular ejection fraction, *LVMI* left ventricular mass index, *RVEDVI* right ventricular end-diastolic volume index, *RVESVI* right ventricular end-systolic volume index, *RVEF* right ventricular ejection fraction, *LGE* late gadolinium enhancement, *ACEI* angiotensin-converting enzyme inhibitors, *ARB* angiotensin receptor blockers, *CMR* cardiovascular magnetic resonance

For binary variables, the number of patients is stated in the far right column with percentage in brackets. For continuous variables mean and standard deviation are stated in the far right column. Atrial fibrillation and ectopics were determined based on prior history. Smoking including current and history of smoking

### 3.2 Correlation between dual-sequence and dual-bolus techniques

There were 2688 segments in total for all 168 patients using the 16 AHA segments model. Seventy-one segments for stress MBF and 65 segments for rest MBF were excluded due to analysis failure, leading to 2617 segments for stress and 2623 segments for rest. One hundred and forty-eight segments were excluded from MPR as a result, leaving 2540 segments.

**Fig. 5:**
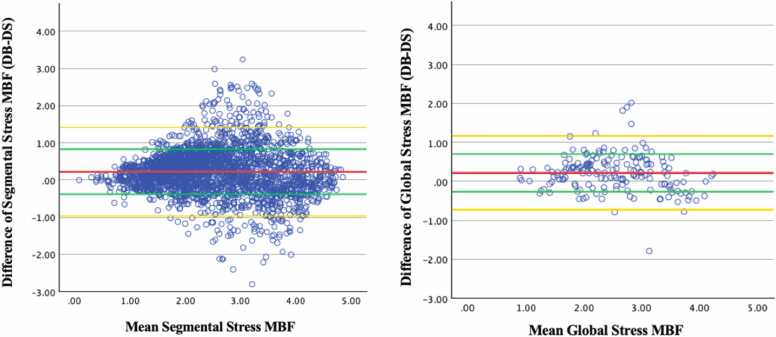
Bland-Altman plots of stress myocardial blood flow (MBF). Segmental stress MBF on the left, followed by global stress MBF on the right. Comparing dual bolus (DB) with dual sequence (DS). Red line indicates average difference of segmental stress MBF (DB-DS); green line indicates 1 standard deviation value away from average difference of segmental stress MBF (DB-DS); yellow line indicates confidence intervals

**Fig. 6:**
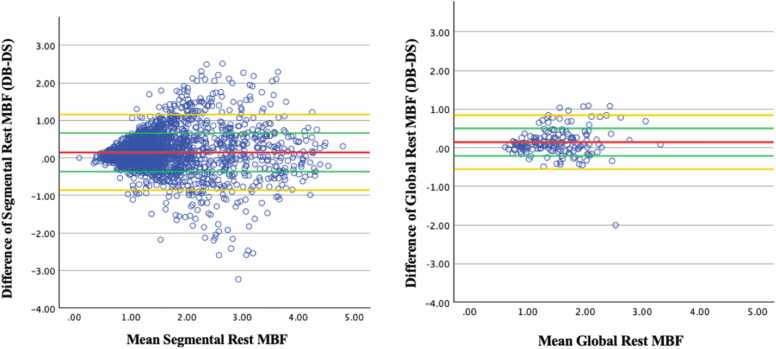
Bland-Altman plots of rest myocardial blood flow (MBF). Segmental rest MBF on the left, followed by global rest MBF on the right. Comparing dual bolus (DB) with dual sequence (DS). Red line indicates average difference of segmental stress MBF (DB-DS); green line indicates 1 standard deviation value away from average difference of segmental stress MBF (DB-DS); yellow line indicates 95% confidence intervals

**Fig. 7:**
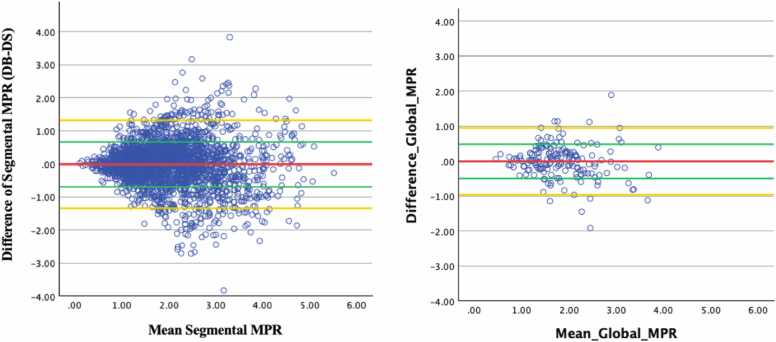
Bland-Altman plots of myocardial perfusion reserve (MPR). Segmental MPR on the left, followed by global MPR on the right. Comparing dual bolus (DB) with dual sequence (DS). Red line indicates average difference of segmental stress MBF (DB-DS); green line indicates 1 standard deviation value away from average difference of segmental stress MBF (DB-DS); yellow line indicates 95% confidence intervals

**Fig. 8:**
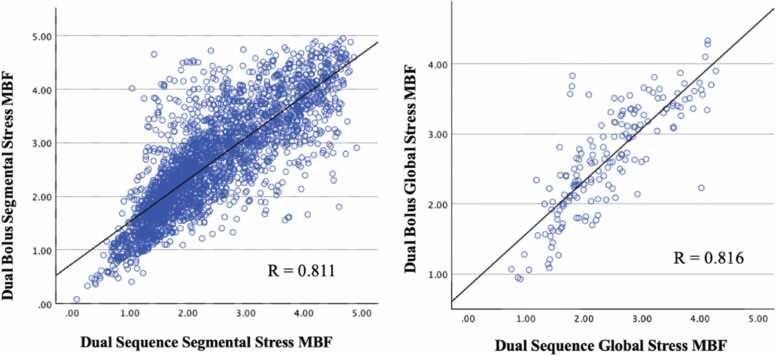
Dual-bolus (DB) vs dual-sequence (DS) stress myocardial blood flow (MBF) scatter plots. Segmental stress MBF on the left, followed by global stress MBF on the right. Correlation of DB stress MBF against DS stress MBF.

**Fig. 9:**
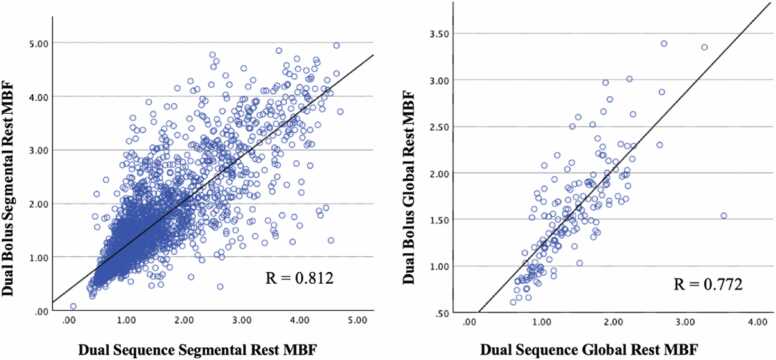
Dual-bolus (DB) vs dual-sequence (DS) rest myocardial blood flow (MBF) scatter plots. Segmental rest MBF on the left, followed by global rest MBF on the right. Correlation of DB rest MBF against DS rest MBF.

**Fig. 10:**
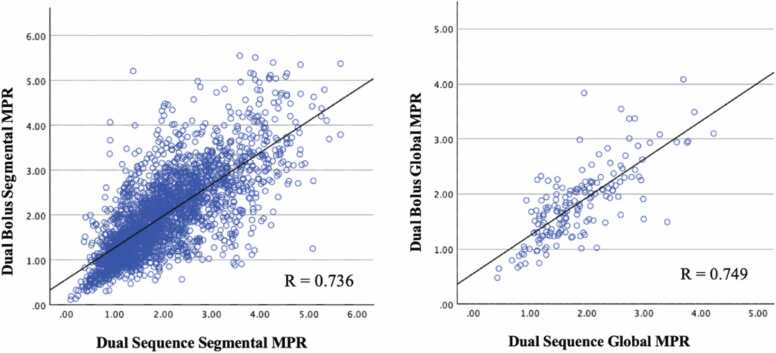
Dual-bolus (DB) vs dual-sequence (DS) myocardial perfusion reserve (MPR) scatter plots. Segmental MPR on the left, followed by global MPR on the right. Correlation of DB MPR against DS MPR

On Bland-Altman analysis, segmental and global stress MBF, DB were 0.22 mL/g/min (±0.61 mL/g/min) and 0.22 mL/g/min (±0.48 mL/g/min) higher than DS on average (Fig. 5). These differences were statistically significant (segmental stress MBF p < 0.001; global stress MBF p = 0.005; Table 3). Difference between segmental and global DB and DS rest MBF was 0.15 ± 0.51 mL/g/min and 0.14 ± 0.36 mL/g/min, respectively (Fig. 6). These differences were statistically significant (segmental rest MBF p < 0.001; global rest MBF p = 0.011; Table 3). Mean difference between DB and DS MPR values was minimal but showed slightly higher standard deviation in both segmental (−0.01 ± 0.68 mL/g/min) and global (−0.01 ± 0.49 mL/g/min) (Fig. 7). The difference was not statistically significant (segmental MPR p = 0.758; global MPR p = 0.773; Table 3). Strong correlations were observed between DB and DS for all MBF and MPR values (range 0.736 to 0.816) as shown in Table 4 and Fig. 8, Fig. 9, Fig. 10.

**Table 3:** Comparison of both dual-bolus and dual-sequence global and segmental quantitative perfusion (QP) parameters.

Parameter	Dual bolus		Dual sequence		p-value
	Median	Interquartile range	Median	Interquartile range	
Segmental stress MBF	2.55	1.46	2.19	1.47	<0.001
Segmental rest MBF	1.32	0.89	1.14	0.77	<0.001
Segmental MPR	1.84	1.27	1.81	1.28	0.758
Global stress MBF	2.63	1.22	2.34	1.12	0.005
Global rest MBF	1.54	0.69	1.37	0.74	0.011
Global MPR	1.77	0.87	1.71	0.95	0.773

*MBF* myocardial blood flow, *MPR* myocardial perfusion reserve

Note: MBF values are in ml/g/min. Interquartile range is the value of the third quartile minus the first quartile.

**Table 4:** Pearson’s correlation values between dual bolus and dual sequence sorted by QP parameter.

	QP parameter	r-value	p-value
Segmental	Stress MBF	0.811	<0.001
	Rest MBF	0.812	<0.001
	MPR	0.736	<0.001
Global	Stress MBF	0.816	<0.001
	Rest MBF	0.772	<0.001
	MPR	0.749	<0.001

*MBF* myocardial blood flow, *MPR* myocardial perfusion reserve, *QP* quantitative perfusion

### 3.3 Evaluation of wall thickness and quantitative perfusion discrepancies

Wall thickness of 168 patients was measured and analyzed. Pearson’s correlation analysis was performed to investigate for correlation between difference in global MBF and MPR values (DB-DS) with wall thickness at different slices and average wall thickness. This assessment did not show any significance between wall thickness of basal slice, mid-ventricular slice, apical slice and average thickness with the difference between global DB and DS for the three QP parameters (Table 5).

**Table 5:** Pearson’s correlation coefficients for wall thickness according to slice with difference between global dual-bolus and dual-sequence quantitative perfusion (QP) parameters.

Wall thickness	Difference in global stress MBF		Difference in global rest MBF		Difference in global MPR	
	r-value	p-value	r-value	p-value	r-value	p-value
Basal slice	0.171	0.027	−0.003	0.971	0.102	0.190
Mid-ventricular slice	0.044	0.569	−0.053	0.493	0.082	0.291
Apical slice	−0.034	0.664	−0.032	0.679	0.021	0.788
Average thickness	0.114	0.142	−0.034	0.658	0.104	0.178

*MBF* myocardial blood flow*, MPR* myocardial perfusion reserve

Note: MBF values are in ml/g/min

### 3.4 Evaluation of quantitative perfusion differences with CMR parameters

Stress heart rate was shown to have very weak but statistically significant correlation between the differences in DB- and DS-derived measurements for rest MBF and MPR (Table 6). Other CMR parameters such as rest heart rate, LV end-diastolic volume indexed (LVEDVI), LV end-systolic volume indexed (LVESVI), LV mass indexed (LVMI), RV end-diastolic volume indexed (RVEDVI), RV end-systolic volume indexed (RVESVI), and RV ejection fraction (RVEF) showed little to no significant correlation with differences between DB and DS QP results.

**Table 6:** Pearson’s correlation coefficients for difference between stress myocardial blood flow (MBF), rest MBF, and myocardial perfusion reserve (MPR) (DB-DS) with CMR parameters and BMI.

CMR parameters and BMI	Difference in global stress MBF		Difference in global rest MBF		Difference in global MPR	
	r-value	p-value	r-value	p-value	r-value	p-value
BMI	−0.009	0.903	0.064	0.407	−0.033	0.67
Stress heart rate	−0.149	0.054	0.238*	0.002*	0.283*	<0.001*
Rest heart rate	−0.109	0.158	0.138	0.074	−0.147	0.057
LVEDVI	−0.079	0.306	−0.09	0.248	0.019	0.802
LVESVI	−0.103	0.182	−0.09	0.248	0.007	0.93
LVSVI	0.026	0.736	−0.021	0.792	0.028	0.723
LVEF	0.095	0.223	0.055	0.477	0.021	0.784
LVMI	−0.051	0.513	−0.05	0.517	−0.005	0.949
RVEDVI	0.056	0.47	−0.03	0.7	0.027	0.728
RVESVI	0.079	0.306	−0.018	0.821	0.021	0.788
RVEF	−0.091	0.243	0.006	0.936	−0.03	0.7

*CMR cardiovascular magnetic resonance, BMI* body mass index*, LVEDVI* left ventricular end-diastolic volume index*, LVESVI* left ventricular end-systolic volume index*, LVSVI* left ventricular stroke volume index*, LVEF* left ventricular ejection fraction*, LVMI* left ventricular mass index*, RVEDVI* right ventricular end-diastolic volume index*, RVESVI* right ventricular end-systolic volume index*, RVEF* right ventricular ejection fraction*, DB* dual bolus*, DS* dual sequence

*= statistically significant

### 3.5 Comparison of ischemic and non-ischemic segments

For both DB- and DS-derived stress MBF and MPR, there were significant differences in the ischemic and non-ischemic segments (all p < 0.0001; Table 7). DB- and DS-derived MBF ischemic and non-ischemic segments did not show statistically significant differences (p = 0.07 and p = 0.14, respectively).

**Table 7:** Comparison of dual-bolus (DB) and dual-sequence (DS) derived stress myocardial blood flow (MBF), rest MBF, and myocardial perfusion reserve (MPR) in ischemic and non-ischemic segments.

	Ischemic segments	Non-ischemic segments	p-value
DB stress MBF	1.66 (1.01–2.54)	2.63 (1.95–3.42)	<0.0001
DS stress MBF	1.44 (1.01–2.11)	2.27 (1.68–3.19)	<0.0001
DB MPR	1.21 (0.81–1.96)	1.89 (1.31–2.59)	<0.0001
DS MPR	1.28 (0.84–1.79)	1.85 (1.30–2.59)	<0.0001
DB rest MBF	1.26 (0.98–1.62)	1.35 (0.97–1.93)	0.07
DS rest MBF	1.12 (0.89–1.43)	1.15 (0.87–1.70)	0.14

Note: MBF values are in ml/g/min

### 3.6 Interobserver and intraobserver variability

A very good interobserver intraclass correlation coefficient of 0.865 was observed when comparing stress MBF and rest MBF values between 2 independent operators using the 16 AHA segment model in QP. An intraobserver ICC of 0.964 was observed from the first operator, whereas the second operator recorded an intraobserver ICC of 0.906.

## 4 Discussion

In our study, we found that DB segmental and global MBF values were significantly higher than DS MBF values for both stress and rest, with a small mean difference of 0.2 mL/g/min observed. MPR demonstrated minimal mean difference which was not statistically significant but slightly higher standard deviations. There were strong correlations between DB and DS MBF and MPR values. In addition, patient factors such as wall thickness, resting heart rate, and LV volumes or ejection fraction did not explain the differences between the two techniques. Stress heart rate was the only factor that showed significance in creating disparities between DB and DS global rest MBF and MPR, but the r-value suggested that there is a very weak correlation. Therefore, this is likely to indicate that differences between DB and DS are likely intrinsic to the techniques themselves. To the best of our knowledge, this is the first study that compares both DB and DS techniques for quantitative stress perfusion to generate stress/rest MBF and MPR.

Many early studies using animal models have validated DB as the standard clinical protocol due to its ability to accurately measure absolute MBF at a larger range of flow rates under ideal situations and proper protocol [4], [5]. DB has also been extensively used in a variety of populations, including the pediatric group [6]. However, many have acknowledged the disadvantages of DB. It is more laborious hence more error-prone, restricting its inclusion in clinical settings [5], [7], [8]. Furthermore, it is difficult to ensure consistency of AIF and myocardial perfusion acquisition due to the gap between bolus injections [9]. Hence in our article, we are addressing the potential of the quantification of cardiac perfusion studies using DS which is a single-bolus injection scheme and much easier to implement.

DS is a more convenient quantitative stress CMR method and is increasingly being adopted into clinical practice due to its simple workflow [11], [12], [13], [14]. There has also been evidence that DS MBF values were in good agreement with positron emission tomography (PET) results [12], [15], [16], [17]. However, some studies using DS with dynamic contrast-enhanced MRI method observed underestimation of PET perfusion ratios [17]. The main concern with DS using Fermi modeling is the oversaturation of AIF due to the high contrast concentration, which may render the quality of MBF and MPR analysis unreliable [8], [18]. There is also a need for software that can efficiently and accurately correct T2* effects to prevent overestimation of MBF [8], [19]. Although the intrinsic differences between DB and DS cannot be ignored, there has been evidence of good agreement between DB and single-bolus methods (without DS) to calculate MPR [20]. Our study also showed no significant difference between average MPR between DB and DS methods, but it showed a slightly higher standard deviation. As MPR is derived from stress MBF divided by rest MBF, this slightly larger standard deviation is likely due to the small differences in stress MBF and rest MBF being amplified when dividing stress MBF by rest MBF. A study in 2015 by Papanastasiou et al. with eight patients also compared DB and single-bolus methods (without DS) against distributed parameter and Fermi modeling techniques [21]. Fermi modeling was found to be more dependent on AIF saturation and significantly overestimated MBF in single-bolus analysis. This is different from our study's results and can likely be explained by the lack of a low resolution with short-saturation preparation time slice being acquired in Papanastasiou et al.’s study [21]. The lack of low resolution with short-saturation preparation time slice means the saturation or blunting of T1 signal intensity to precisely calculate AIF cannot be overcome in Papanastasiou et al.’s study [21] and thus would result in higher MBF values.

A study with 15 patients, by Gatehouse et al., investigated the accuracy of AIF of 3 different CMR methods: low-dose injection with high T1-sensitivity sequence (LDHT, DB), high-dose injection with low T1-sensitivity sequence (HDLT, DS), and high-dose injection with high T1-sensitivity sequence [8]. The AIF baseline values of LDHT (dual bolus) rest and stress were both slightly higher than HDLT (dual-sequence), but the peak values of region of interest were similar. These differences are caused by different doses and T1 sensitivities between DB and DS [8], [9], [22]. Subsequently, the method with AIF of a higher baseline value may lead to lower MBF values. This occurrence may explain our results of higher DB MBF values compared to DS. It is also important to note that although this Gatehouse et al. study conducted rest perfusion sequence before stress, which might overestimate baseline stress AIF values due to residual gadolinium contrast, the rest perfusion images for AIF already showed minor elevation in DB compared to DS. In addition, it is possible the DS sequence is not fully optimized to avoid T2* effects, and therefore potentially limits the dynamic range of the AIF due to saturation of the signal.

Results from this study ultimately suggest that direct comparisons of QP analysis across DB and DS techniques should be done with caution, particularly with stress and rest MBF. Minor variances can skew the interpretation of a patient’s myocardial perfusion and influence medical decision-makings. It is recommended that stress CMR imaging protocols be standardized to either one of the techniques in a clinical setting to prevent discrepancies in the analysis of QP results for patients. Clinical professionals should be aware of these discrepancies between the two techniques and take this difference into account if one chooses to utilize diagnostic cut-offs to diagnose coronary microvascular dysfunction or triple vessel disease. Careful attention should be paid to whether a DB or DS technique was utilized to determine these cut-offs. Currently, our results do not indicate which technique is more accurate to diagnose obstructive coronary artery disease or its prognostic significance. Additionally, for future research studies adopting QP methods, it is also advised to utilize one technique if comparing values across different periods. This is especially important in cases comparing patient groups or drug effect/intervention on myocardial perfusion, as minor fluctuations caused by different image acquisition techniques could be misinterpreted as the effect of the intervention.

## 5 Limitations

The main strength of our study is that both the DB and DS methods are conducted on the same patient simultaneously using the same scanner and same contrast bolus injection, eliminating potential errors or confounders such as different hemodynamic responses to adenosine on separate occasions. However, this is also a limitation as further studies need to be performed on other scanners and different patient/volunteer populations. In addition, patients with heart transplants and congenital heart disease were not included in this cohort, so these results may not be generalizable to these populations. Our study also did not compare repeated measurements within the same patient using either DS or DB to demonstrate the interscan differences as a reference. Furthermore, this is a retrospective study and prospective studies need to be conducted to confirm our findings. For this DB and DS technique, there is also a lack of validation for clinical coronary artery disease. In addition, the DS protocol will inevitably have a slightly higher contrast dose than normal. However, this difference is minor and thought to not cause any significant changes to our overall results.

## 6 Conclusion

DB and DS methods in CMR image acquisition produce quantitative myocardial perfusion results that show strong correlation. However, DB MBF values are significantly higher on average than DS MBF values while DB and DS MPR values are not significantly different but with slightly higher variability between the two techniques. Caution should be taken when comparing studies using DB techniques vs studies using DS techniques to prevent misinterpretation of data.

## Funding

Not applicable.

## Author contributions

**Emily Yin Sing Chong**: Investigation, Data curation, Formal analysis, Writing – original draft, Writing – review and editing. **Haonan Wang**: Data curation, Software, Supervision, Writing – original draft, Writing – review and editing. **Kwan Ho Gordon Leung, Tsun Hei Sin**: Data curation, Software, Writing – review and editing. **Victor Goh**: Data curation, Writing – review and editing. **Paul Kim, Yuko Tada**: Methodology, Writing – review and editing. **Chun Ka Wong, Chor Cheung Frankie Tam, Mitchel Benovoy, Andrew E. Arai, Martin A. Janich, Amit R. Patel** : Writing – review and editing. **Ming-Yen Ng**: Conceptualization, Methodology, Data curation, Writing – original draft, Writing – review and editing, Project administration.

## Ethics approval and consent to participate

The study was approved by the Hong Kong West Cluster Institutional Review Board. Ethics approval number: UW24-197. Patient consent was waived due to the study being a retrospective study.

## Consent for publication

Not applicable.

## Declaration of competing interests

The authors declare the following financial interests/personal relationships which may be considered as potential competing interests: Ming-Yen Ng reports a relationship with Bayer AG that includes funding grants and speaking and lecture fees. Ming-Yen Ng reports a relationship with GE Healthcare that includes funding grants and speaking and lecture fees. Ming-Yen Ng reports a relationship with Circle Cardiovascular Imaging Inc. that includes funding grants and speaking and lecture fees. Ming-Yen Ng reports a relationship with Boerhinger Ingelheim that includes speaking and lecture fees. Ming-Yen Ng reports a relationship with Lode B.V. that includes funding grants. Ming-Yen Ng reports a relationship with Arterys Inc. that includes funding grants. Ming-Yen Ng reports a relationship with TeraRecon Inc. that includes funding grants. Associate Editor of JCMR - Ming-Yen Ng and Amit Patel; Associate Editor of JCCT - Ming-Yen Ng. Haonan Wang and Martin Janich are employees of GE HealthCare. Amit Patel has received research grants from GE Healthcare and research support from CircleCVI, Neosoft, and Siemens Healthineers. Mitchel Benovoy is the Chief Executive Officer of Area19 Medical, the Chief Executive Officer of ViTAA Medical Solutions, and a Member of The Board of Advisors at Yunu, Inc. Andrew E Arai has received royalty payments from Circle CVI. The other authors declare that they have no known competing financial interests or personal relationships that could have appeared to influence the work reported in this paper.
